# The Screening of Broadly Neutralizing Antibodies Targeting the SARS-CoV-2 Spike Protein by mRNA Immunization in Mice

**DOI:** 10.3390/pharmaceutics15051412

**Published:** 2023-05-05

**Authors:** Zhiyin An, Yu Zhang, Xiang Yu, Jia Xia, Yanan Yin, Guoming Li, Jing Lu, Xuemei Fan, Yingjie Xu

**Affiliations:** 1 Department of Biochemistry and Molecular Cell Biology, Shanghai Key Laboratory for Tumor Microenvironment and Inflammation, Shanghai Jiao Tong University School of Medicine, Shanghai 200025, China; anzhiyinxiaohuihui@sjtu.edu.cn (Z.A.);; 2 Department of Nephrology, Renji Hospital, School of Medicine, Shanghai Jiao Tong University, Shanghai 200127, China; 3 Department of Pathophysiology, Shanghai Jiao Tong University School of Medicine, Shanghai 200025, China; 4 Key Laboratory of Cell Differentiation and Apoptosis of Chinese Ministry of Education, Shanghai Jiao Tong University School of Medicine, Shanghai 200025, China; 5 Shanghai RNACure Biopharma Co., Ltd., Shanghai 200438, China

**Keywords:** SARS-CoV-2, spike, mRNA vaccine, broadly neutralizing antibody

## Abstract

Neutralizing antibodies (nAbs), the popular antiviral drugs used for the treatment of COVID-19, are effective in reducing viral load and hospitalization. Currently, most nAbs are screened from convalescent or vaccinated individuals through single B-cell sequencing which requires cutting-edge facilities. Moreover, owing to the rapid mutation of SARS-CoV-2, some approved nAbs are no longer effective. In the present study, we designed a new approach to acquiring broadly neutralizing antibodies (bnAbs) from mRNA-vaccinated mice. Using the flexibility and speed of mRNA vaccine preparation, we designed a chimeric mRNA vaccine and sequential immunization strategies to acquire bnAbs in mice within a short period. By comparing different vaccination orders, we found that the initially administered vaccine had a greater effect on the neutralizing potency of mouse sera. Ultimately, we screened a strain of bnAb that neutralized wild-type, Beta, and Delta SARS-CoV-2 pseudoviruses. We synthesized the mRNAs of the heavy and light chains of this antibody and verified its neutralizing potency. This study developed a new strategy to screen for bnAbs in mRNA-vaccinated mice and identified a more effective immunization strategy for inducing bnAbs, providing valuable insights for future antibody drug development.

## 1. Introduction

The past three years have been dominated by the COVID-19 pandemic which has led to over six million deaths worldwide [1]. The pandemic was caused by SARS-CoV-2, a highly pathogenic coronavirus, attaining widespread prevalence; this followed the pandemics caused by SARS-CoV in 2002 and MERS-CoV in 2012 [2,3,4]. With the emergence of various variants, including the Alpha, Beta, Delta, and Omicron variants, the global health situation has become increasingly complex.

The COVID-19 pandemic has led to the rapid development of vaccines, particularly mRNA vaccines [5,6]. The mRNA vaccines have several advantages, including short development cycles, the ease of transcription, and the ability to respond quickly to new variants [6,7]. However, a significant reduction in antibody titers was observed in immunocompromised patients compared with immunocompetent individuals after vaccination [8]. A potential risk of illness also exists in individuals with high antibody titers because of the immune evasion of emerging variants [9,10]. Therefore, despite the increasing vaccinations, breakthrough infections still occur, and achieving global vaccination remains a complex challenge [11,12].

Presently, most vaccines and drugs for COVID-19, including neutralizing antibodies (nAbs), target the spike protein of SARS-CoV-2 which anchors the coronavirus membrane and recognizes the receptor angiotensin-converting enzyme 2 (ACE2) [13,14,15,16,17]. nAbs are used to prevent infection in high-risk individuals and treat patients with suppressed immune systems [9,18,19,20,21,22,23,24,25]. During the pandemic, several nAb drugs, including monoclonal antibodies (mAbs) and antibody cocktails were approved by the Food and Drug Administration (FDA) to mitigate COVID-19 symptoms [26]. Of these, several recently developed antibodies with broad-spectrum neutralizing potencies received emergency use authorization for their effectiveness [26]. However, mutations in the antibody-binding sites on the spike proteins of SARS-CoV-2 variants of concern (VOCs) cause resistance to existing nAbs [27,28,29]. The emergence and prevalence of the Omicron BA.1 variant, with over 30 mutations on its spike protein, have led to a pause in the use of several approved antibody drugs [30,31,32,33].

Broadly neutralizing antibodies (bnAbs) target a relatively conserved region of the spike protein, making them less affected by frequent mutations and allowing them to retain their neutralizing potency against multiple variants [34,35,36]. To date, all approved bnAbs against SARS-CoV-2 have been obtained from clinical resources that rely on convalescent patients or vaccinated individuals and timely strategies for producing these antibodies in the laboratory are needed [37].

Our previous study showed that mRNA vaccination elicited strong humoral and cellular immune responses in mice [38]. In the present study, we aimed to develop an efficient method for producing nAbs in biosafety level II laboratories based on the flexibility and speediness of mRNA vaccination.

## 2. Materials and Methods

### 2.1. Cell Culture and Reagents

The human embryonic kidney cell line (HEK 293T) was used to evaluate the levels of proteins expressed by transfected mRNAs or plasmids and to construct stable cell lines, which were maintained in high-glucose Dulbecco’s modified Eagle’s medium (DMEM, L110KJ; Basalmedia, Shanghai, China) supplemented with 10% fetal bovine serum (FBS, 10500064; Gibco, Carlsbad, CA, USA). SP2/0-Ag14 cells were used in the hybridoma technology and maintained in RPMI 1640 medium (L210KJ; Basalmedia, Shanghai, China) that was supplemented with 20% fetal bovine serum (FBS; Sigma Aldrich, Burlington, MA, USA). Hybridoma cells were cultured in HAT medium, which is RPMI 1640 supplemented with 20% fetal bovine serum, 1% mixture of streptomycin and penicillin (S110JV; Basalmedia, Shanghai, China), 1% L-glutamine (G7513; Sigma-Aldrich, Burlington, MA, USA), 1% non-essential amino acid (11140050; Thermo Fisher Scientific, Waltham, MA, USA), 1 × hypoxanthine-aminopterin-thymidine (HAT, H0262; Sigma-Aldrich, Burlington, MA, USA), and 1 × hybridoma feeder (MAC0014; Frdbio, Wuhan, Hubei, China).

All cells were purchased from American type culture collection and were cultured in a humidified incubator with 5% CO_2_ at 37 °C and were verified to be free of mycoplasma.

### 2.2. Plasmid Construction

A codon-optimized SARS-CoV-2 spike gene from the Wuhan-Hu-1 (wild-type) strain, including the D614G mutation, which is truncated by 18 amino acids at the C-terminus, was cloned into the pCAG plasmid. This plasmid was used as a template to construct variant spike expression vectors. Mutants of the variant spikes were introduced using specific primers (Table S1). The vector was linearized using Phusion High-Fidelity DNA Polymerase (K1030; APExBIO, Houston, TX, USA) and verified by electrophoresis on a 1% agarose gel. The target bands were collected using GeneJET Gel Extraction Kit (K0692; Thermo Fisher Scientific, Waltham, MA, USA). After digestion with Dpn1 (R0176S; NEB, Ipswich, MA, USA), the linearized products were ligated by Gibson assembly using a ClonExpress II One Step Cloning Kit (C112; Vazyme, Nanjing, Jiangsu, China). Plasmids were extracted from single colonies that were amplified using the Qiagen Plus Midi kit (12945; QIAGEN, Hilden, Germany).

### 2.3. Stable Cell Line Construction

The lentivirus particles used for cell infection were produced by transfecting HEK293T cells with a plasmid (plenti-hACE2) and a mixture of packaging vectors (Tet2; MPMG; Rev; VSV-G) using polyethylenimine (PEI, 23966-1; PolyScience, Philadelphia, PE, USA). After 48 h, the cell culture supernatant containing lentivirus particles was filtered and used for subsequent infection with the aid of polybrene (8 μg/mL). Following 48 h of infection, the cells were screened with 1 μg/mL puromycin (B7587; APExBIO, Houston, TX, USA) for 3–5 days to obtain positive cells. To ensure experimental reproducibility, multiclonal 293T hACE2 cells were seeded into 96-well plates at a limited dilution for cloning. Single colonies were screened for hACE2 expression using western blot (WB) analysis (Figure S1a). Cell lines showing high expression levels were selected for further investigation.

### 2.4. Preparation of Modified mRNA

For mouse immunization, spike variant mRNAs were transcribed in vitro using T7 polymerase, as previously described [38,39]. In brief, the codon-optimized open reading frame (ORF) that was flanked with the 5′ and 3′ untranslated regions was synthesized by General Biology (Chuzhou, Anhui, China) and then cloned into the pcDNA3.1 vector with a T7 promoter upstream. DNA templates for mRNA synthesis were prepared by a polymerase chain reaction (PCR) using the synthesized primers with the reverse strand containing poly (T) that were purchased from Genscript (Nanjing, Jiangsu, China). A Cap1 analog EZ Cap^TM^ Reagent AG (B8176; APExBIO, Houston, TX, USA) was placed at the 5′ ends of the mRNAs. To avoid non-specific immunoreactions caused by non-modified mRNAs, the uridines used for in vitro transcription (IVT) were replaced with N1-methyl-pseudouridine (m^1^φ) or pseudouridine (φ) [40]. Thereafter, the mRNAs were then purified using an RNA Clean and Concentrator Kit (R1018; ZYMO Research, Irvine, CA, USA).

### 2.5. Packaging of mRNA-Loaded Lipid Nanoparticles (LNPs)

The formulations for mRNA-loaded lipid nanoparticles (LNPs) have been described in a previous study [38]. Briefly, DLin-MC3-MDA, distearylphosphoryladenocholine (DSPC), cholesterol, and PEG2000-DMG were dissolved in ethanol in the ratio of 50:10:38.5:1.5 and mixed with an equal volume of mRNA in a 50 mM citrate solution using a T-mixer. The mixture was immediately diluted 2-fold with 50 mM citrate buffer (pH 3.0). The resulting formulation was then dialyzed in Dulbecco’s phosphate-buffered saline (DPBS, pH 7.4) using slide-a-lyzer dialysis cassettes (66380; Thermo Fisher Scientific, Waltham, MA, USA) for at least 15 h. This was followed by concentration of the formulation using Amicon Ultra Centrifugal Filters (UFC80030; Millipore, Billerica, MA, USA) and filtration through a 0.22-μm filter (SLGP033RB; Millipore, Billerica, MA, USA). The encapsulation rates of mRNAs were determined using a Quant-iT RiboGreen RNA assay (R11490; Thermo Fisher Scientific, Waltham, MA, USA). The particle sizes were determined using a Zetasizer Nano ZS (Malvern, UK).

### 2.6. Transfection of mRNAs and Expression Vectors

Prior to mouse immunization, the expression of mRNA synthesized in vitro were evaluated. For this, 2 μg of mRNAs per well (six-well plate) were transfected into 293T cells with ~70% confluence using lipofectamine 2000 (11668019; Thermo Fisher Scientific, Waltham, MA, USA) in the mass–volume ratio of 1:2. For the expression vectors, 2 μg per well were transfected into 293T cells at 70% confluence using 6 μL polyethylenimine (PEI, Polyscience, Philadelphia, PE, USA). Alternatively, 2 μg of mRNA packed in lipid nanoparticles are added directly to the wells. The mass ratio of heavy chain and light chain for transfection is 2.7:1 for antibody mRNAs and 1.5:1 for the antibody expression vectors.

### 2.7. Membrane Fusion Imaging

To verify the function of the mutant spikes in the constructed expression vectors, we performed a membrane fusion experiment mediated by the spikes and hACE2 was performed. Different spike construct plasmids were transfected into hACE2 overexpressed 293T cells. After 36 h, the cells were washed with DPBS, stained with 2 μM Hoechst 33,342 (62249; Thermo Fisher Scientific, Waltham, MA, USA) at 37 °C for 15 min, and washed three times with DPBS for microscopic observation and image acquisition using a fluorescence microscope (Leica DMi8; Weztlar, Germany).

### 2.8. Western Blot Analysis

Cells transfected with plasmids or mRNA were collected and lysed in mammalian cell lysis buffer (MCLB) which was composed of 50 mM pH 7.5 Tris, 150 mM NaCl, and 0.5% IGEPAL^®^ CA-630 (I3021; Sigma-Aldrich, Burlington, MA, USA) completed with an EDTA-free protease inhibitor cocktail (04693159001; Roche, Basel, Switzerland) and 1 mM PMSF (0754; Amresco, Solon, OH, USA) on ice for 30 min and centrifuged at 12,000 rpm for 20 min at 4 °C. The supernatant was collected, and the protein concentrations were determined using the Quick Start Bradford assay (5000201; Bio-Rad, Hercules, CA, USA). About 15 or 25 μg of protein was electrophoresed with 15-well or 10-well 4–20% gradient sodium dodecyl sulfate polyacrylamide gels at 90 to 120 V stable voltage and transferred to nitrocellulose filter membranes (Millipore, Billerica, MA, USA). After being blocked with 5% skim milk (Sangon Biotech, Shanghai, China) in Tris-buffered saline with 0.1% Tween-20 (150 mM NaCl and 50 mM Tris–HCl at pH 7.4 and 0.1% Tween-20), the membranes were incubated with corresponding primary antibodies overnight at 4 °C. The expression of specific proteins was determined using a ChampChemi imaging system (Sage Creation Science, Beijing, China) after incubation with horseradish peroxidase (HRP)-conjugated goat anti-mouse IgG (H+L) (SA00001-1; Proteintech, Wuhan, China). The antibodies used for WB included the 2019n-CoV antibody (40589-T62; Sinobiological, Beijing, China), the mouse β-actin antibody (66009; Proteintech, Wuhan, China), and the rabbit-ACE2 antibody (4355; Cell Signaling technology, Danvers, MA, USA).

### 2.9. Cryo-TEM Sample Preparation and Imaging

A glow-discharged grid (R2/1 Cu, 300 mesh; Quantifoil, Großlöbichau, Germany) was used to apply the LNP samples. After blotting at 100% humidity and 4 °C for 3 s, the grids were immersed in liquid ethane in a Mark IV vitrobot (Thermo Fisher Scientific, Waltham, MA, USA). Images were acquired in the low-dose mode using a Talos F200C G2 transmission electron microscope (Thermo Fisher Scientific, Waltham, MA, USA). The pixel size was set to 5.75 Å at a magnification of 36,000×.

### 2.10. Animals

Female BALB/c mice aged 6–8 weeks were purchased from SLAC Subsidiary Corporation (Shanghai, China) and were used for immunization, blood collection, and splenocytes extraction. All animal experiments were conducted in strictly regulated and pathogen-free facilities at the Shanghai Jiao Tong University School of Medicine. The Institutional Animal Care and Use Committees of Shanghai Jiao Tong University School of Medicine approved all animal procedures.

### 2.11. Animal Experiment

The BALB/c mice were divided into different experimental groups, including a negative control (NC) group. Orbital blood was collected after two doses of immunization and antibody titers were determined using an enzyme-linked immunosorbent assay (ELISA). The neutralizing potency of the mouse sera was compared after three or four immunization doses. The mice with the highest antibody titers were selected for hybridoma production.

### 2.12. ELISA

To detect antibody titration in mouse sera, hybridoma supernatant and the medium of 293T cells were transfected with antibody expression vectors. Ninety-six-well binding plates (442402; Thermo Fisher Scientific, Waltham, MA, USA) were incubated with 100 μL of 1 μg/mL spike protein (40589-V08B1; sinobiological, Beijing, China) overnight at 4 °C. The following day, the plates were blocked with 100 μL of blocking buffer (2% BSA, 36101ES60; Yeasen, Shanghai, China, DPBS, 21600-044; Gibco, Carlsbad, CA, USA) for 1 h at room temperature after being washed with 200 μL PBST (PBS, 0.05% Tween-20). Serially diluted immunized serum samples or cell media were then incubated in the plates for 2 h at room temperature followed by incubation with a 1:5000 HRP-conjugated goat anti-mouse IgG(H+L) (SA00001-1; Proteintech, Wuhan, China) for 1 h. For detection, 50 μL 3,3′,5,5′-tetramethylbenzidine (TMB, 00-4201-56; Invitrogen, Waltham, MA, USA) was added to each well and incubated for 5–10 min. The colorimetric reaction was terminated using a stop buffer (C1058; SolarBio, Beijing, China) which was followed by OD450 detection using a microplate reader (Infinite 200 PRO, Tecan, Männedorf, Switzerland).

### 2.13. Pseudotyped VSV-SARS-CoV-2 Spike Construction

SARS-CoV-2 Fluc B.1.1.529 (dd1568-01; Vazyme, Nanjing, Jiangsu, China) and 293T ACE2 cells were used to prepare VSV-△G-luciferase G which was used to produce SARS-CoV-2 pseudoviruses [41]. The plasmids encoding variant spikes were verified to be well expressed in 293T cells by WB before being used for pseudovirus production (Figure S2a). Ten micrograms of plasmids encoding the VSV-G protein or variant spikes were transfected into 293T ACE2 cells or 293T cells at 60–70% confluency 24 h prior to infection with SARS-CoV-2 Fluc B.1.1.529 or VSV-△G-luciferase G (Figure S2b). At 1.5 h after infection, the medium was removed, and the cells were gently rinsed with serum-free DMEM. Then, 293T ACE2 cells were replenished with complete DMEM, whereas 293T cells were replenished with 1:300 anti-VSV-G rat serum DMEM containing 5% FBS. After 48 h, the supernatants were collected and filtered using a sterile 0.45 μm syringe filter (SLHVM33RS; Millipore, Burlington, MA, USA).

To determine the titration of pseudotyped virus, 293T ACE2 cells seeded in white 96-well plates were infected with 3-fold serially diluted pseudotyped virus. After 24 h, the media were removed, and 100 μL D-luciferin solution was added (1 mM D-luciferin, 122799; PerkinElmer, Waltham, MA, USA, 2 mM ATP·Na_2_, A600020-0005; Sangon, Shanghai, China, 1 mM PMSF dissolved in MCLB). After 10 min of this addition, the fluorescence signals were captured using a microplate reader (Infinite 200 PRO, Tecan, Männedorf, Switzerland). Commercial SARS-CoV-2 Fluc B.1.1.529 with a concentration of 1.3 × 10^4^/mL TCID_50_ was used for standardization. The dilution time for each pseudovirus was interpolated by fitting a three-parameter curve using GraphPad Prism 9.0 (Figure S2c).

### 2.14. Neutralizing Assay

The 100 × initially diluted mouse sera were three-fold serially diluted and mixed with 50 μL 1.3 × 10^4^/mL TCID_50_ pseudotyped virus and incubated at 37 °C for 1 h. Afterwards, 40,000 293T hACE2 cells were seeded in 100 μL each well. After incubation of mice sera with pseudoviruses, the nAbs in the sera bind to the spike protein on the envelope of the pseudovirsuses, blocking the cell entry of SARS-CoV-2 pseudovirus to 293T hACE2 cells (Figure S2d). A total of 24 h later, the media were replaced with 100 μL per well of D-luciferin solution. The plate was then shaken for 10 min at room temperature and the fluorescence signal was measured using a microplate reader (Infinite 200 PRO, Tecan, Männedorf, Switzerland). The relative light units (RLU) of each well were negatively correlated with the nAb titers in the serum. The inhibition or neutralization rate was calculated based on the RLU reduction in experimental wells compared to viral control wells without any serum sample.

### 2.15. Hybridoma Technology

SP2/0 cells were prepared, and the immunized mice were boosted with 6 μg mRNA LNP through the tail vein 3 days before sacrifice. All splenocytes were collected and fused to the same number of SP2/0 cells using 50% PEG (P7306; Sigma-Aldrich, Burlington, MA, USA). The cell number and viability were measured after erythrocyte lysis using ammonium-chloride-potassium (ACK) lysis buffer (3702; Beyotime Biotechnology, Shanghai, China) and trypan blue dye (15250061; Thermo Fisher Scientific, Waltham, MA, USA). The cells were then cultured and selected using hybridoma-specific HAT medium, as described for the cell culture and reagents. The media in all the wells were replaced with fresh HAT medium three days before ELISA detection. Cells in the positive wells were cloned and subcloned into ClonaCell-HY medium D without HAT (3810; Stem Cell, Vancouver, BC, Canada). After the amplification of anti-spike hybridoma cells, the medium was collected to detect the neutralizing potency of the mAbs. Hybridoma cells that secreted bnAbs were collected for antibody sequencing.

### 2.16. Hybridoma Culturing in Mice Ascites

BALB/c mice were administered a single intraperitoneal (i.p.) injection of 0.5 mL pristane (MB0318-1; Bergolin, Dalian, China). After 14 days, the mice were injected with a single i.p. injection of 5 × 10^6^ hybridoma cells in a volume of 0.5 mL, after which they were examined daily for ascites collection following abdominal distention.

### 2.17. mAb Sequencing

To reverse-transcribe the heavy and light chains in monoclonal hybridoma cells, three synthesis reactions were performed. The mRNA of hybridoma cells was extracted using the EZ-press RNA purification kit (B0004DP; EZBioscience, Roseville, MN, USA) and reversed in vitro with reverse RT primers of kappa, lambda, and heavy chains and a universal forward primer containing a template-switch oligo using SMARTScribe reverse transcriptase (639537; Clontech, Mountain View, CA, USA) according to the manufacturer’s instructions. The cDNAs were firstly amplified in low-cycle 5′ rapid amplification of cDNA end (RACE) PCR reaction and then amplified by nested PCR and subcloned into expression vectors containing human antibody constant regions. The primers used for reverse transcription, RACE PCR, and nested PCR are listed in Table S2.

### 2.18. Statistical Analysis

All statistical analyses were performed using GraphPad Prism 9.0. A three-parameter inhibitor vs. response was used for the pNT50 calculation. Differences between the groups were analyzed using Welch’s *t*-tests. Error bars represent the standard deviation (SD). Statistical significance was set at *p <* 0.05 with significance values shown in figures indicated as follows: * *p* < 0.05, ** *p <* 0.01, *** *p* < 0.001, and **** *p* < 0.0001.

## 3. Results

### 3.1. mRNA Antigen Design and Optimization

**Figure 1:**
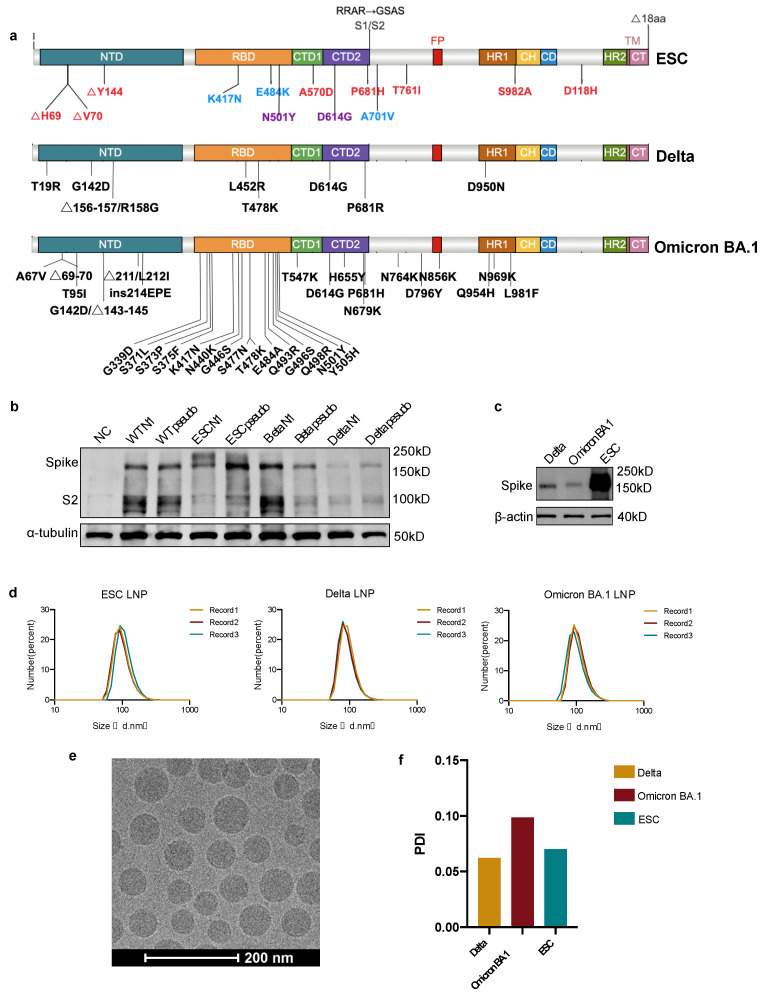
A schematic representation of design, optimization, and preparation of mRNA vaccines. (**a**) Antigen design of England and South Africa Chimera (ESC), Delta and Omicron BA.1 spike vaccines. In the ESC antigen, mutations in the Alpha variant are marked in red, the Beta variant in blue, and shared mutations in purple. The RRAR at the furin-cleavage site was replaced with GSAS. All mutations in Delta and Omicron BA.1 spikes are marked in black. (**b**) Verification of expression of pseudouridine (marked as pseudo) and N1-methyl-pseudouridine (marked as N1) modified mRNAs in HEK293T cells by western blot (WB) analysis. (**c**) Verification of expression of Delta, Omicron BA.1, and ESC mRNAs in HEK293T cells by WB which were used for mice vaccination. (**d**) The distribution of the diameters of lipid nanoparticles. Every sample was detected thrice. (**e**) The structure of mRNA LNPs under the cryo-TEM. Scale bar = 200 nm. (**f**) The polydispersity indexes (PDIs) of Delta, Omicron BA.1, and ESC LNPs. NTD, N-terminal domain; RBD, receptor binding domain; CTD, C-terminal domain; FP, fusion peptide; HR, heptad repeat; CH, central helix; CD, connector domain; CT, cytoplasmic terminal.

To elicit a humoral immune response and acquire bnAbs in mice, we designed an England and South America chimeric antigen (ESC) encoding a near full-length spike protein lacking the 18 cysteine-rich residues at the extreme C-terminus (Figure 1a). To prevent protease-mediated proteolysis, we replaced the furin-cleavage site of _682_RRAR_685_ with _682_GSAS_685_ [42]. Compared to the wild-type spike protein of SARS-CoV-2, this ESC immunogen contains all mutations in the Alpha and Beta variants. Additionally, we designed and synthesized full-length spike mRNAs encoding the spikes of the Delta and the Omicron BA.1 variants to compare different immunization strategies and to produce bnAbs (Figure 1a). Unmodified mRNA induces a strong immune response against molecular mRNA, which will impair the effectiveness of vaccines [43,44]. By comparing the expression levels of the mRNA vaccine with pseudouridine and N1-methyl-pseudouridine modifications by WB, we observed higher expression levels of mRNAs encoding wild-type, ESC, and Delta spikes with pseudouridine modification (Figure 1b). The expression of Delta, Omicron BA.1, and ESC mRNAs was verified before vaccination, where the ESC showed the highest expression levels (Figure 1c). The mRNAs were then encapsulated in LNPs for in vivo delivery and the diameters of most LNPs were close to 100 nm (Figure 1d). Under cryo-TEM, the spherical LNPs were evenly distributed (Figure 1e). The polydispersity index (PDI) of the LNPs was less than 0.10, indicating a monomodal dispersion (Figure 1f).

### 3.2. Different mRNA Vaccination Strategies Elicit Different Titers of Anti-Spike Antibody in Mice Sera

**Figure 2:**
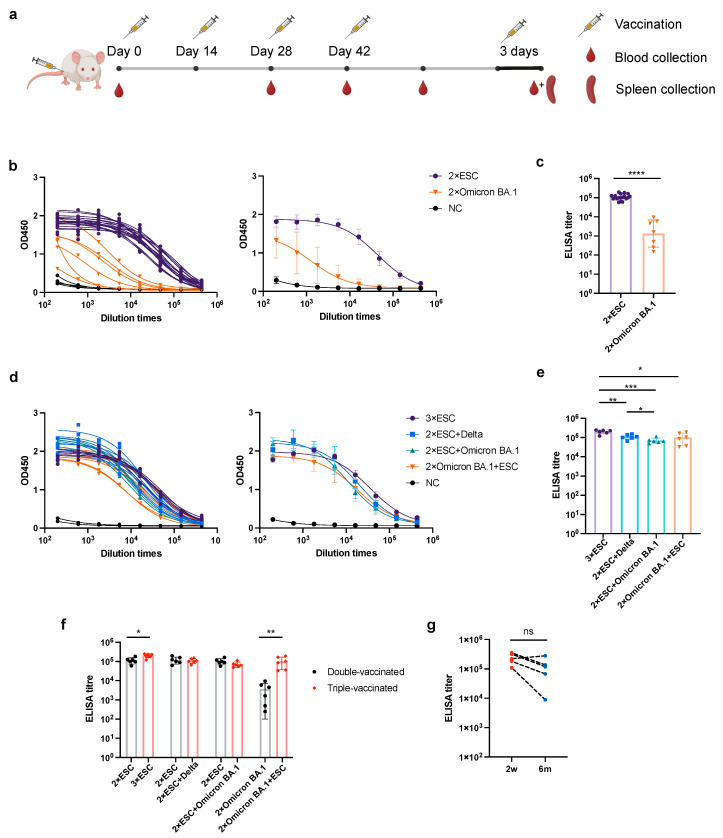
Mouse immunization and antibody titers in different groups. (**a**) Timeline of the mouse immunization. The diagram was created on Biorender.com. (**b**) OD450 values of enzyme-linked immunosorbent assay (ELISA) with sera from double-vaccinated mice during serial dilution. Individual curves are shown on the left, whereas grouped curves are shown on the right. (**c**) ELISA titers of grouped data from double vaccinated sera. (**d**) OD450 values of ELISA with sera from triple-vaccinated mice in serial dilutions. Individual curves from triple-vaccinated sera are shown on the left while grouped curves are shown on the right. (**e**) ELISA titers of grouped data from triple vaccinated sera. (**f**) Comparison of ELISA titers between double- and triple-vaccinated sera in each group. (**g**) Paired comparison of ELISA titers of ESC triple-vaccinated mice sera collected two weeks (2w) and six months (6m) after the final vaccination. The dilution endpoint cutoff was determined using the mean OD450 and threefold SD of the negative control group at the initial dilution, which was 0.557 in (**c**), 0.394 in (**e**), and 0.248 in (**g**). All data are presented as mean ± standard deviation (SD). The difference in the endpoint titer was determined using Welch’s *t* test. * *p* < 0.05, ** *p* < 0.01, *** *p* < 0.001, and **** *p* < 0.0001. *n* = 6.

To investigate the impact of different immunization strategies on the production of anti-spike antibodies and nAbs, we employed homogeneous and heterogeneous immunization strategies in different orders using ESC, Delta, and Omicron BA.1 antigens (Table 1). Group A mice received three doses of ESC, whereas groups B and C received two doses of ESC as a baseline immunization and two doses of Delta or Omicron BA.1 antigens, respectively, as boosts. Group D mice received two doses of the Omicron BA.1 antigen followed by two doses of the ESC antigen. The NC group was treated with empty LNPs. All mice were vaccinated every two weeks with 6μg mRNAs and blood was collected on days 1, 28, 42, and 56 after vaccination (Figure 2a). ELISA results showed that two doses of vaccination induced IgG binding to the wild-type spike protein in mice (Figure 2b). The anti-spike antibody titer of ESC-vaccinated sera was higher than that of Omicron BA.1-vaccinated sera, possibly because the expression of Omicron BA.1 mRNA in vitro was lower than that of ESC, and multiple mutations on the spike of Omicron BA.1 reduced the possibility of inducing anti-wild-type spike antibodies (Figure 2c). After the third boost, the antibody titers in group D were closer to those in the other groups (Figure 2d,e). The effect of a booster immunization was also investigated by comparing the antibody titers of double- and triple-vaccinated sera within the same group. The antibody titers in groups A and D increased dramatically after the third boost with ESC, whereas those in groups B and C did not change after the Delta or Omicron BA.1 boost (Figure 2f). This can be explained by the lower expression levels of Delta and Omicron BA.1 compared with those in ESC. Therefore, we inferred that the expression levels of booster vaccines significantly affected their efficacy. A total of 6 months later, the mouse sera in group A was collected and compared with the sera collected on day 42. The anti-spike antibody titer of mouse sera did not decrease over six months (Figure 2g).

### 3.3. The Prime Vaccine Has More Influence on the Neutralizing Tendency

Owing to the highly pathogenic and infectious nature of SARS-CoV-2 and its variants, handling them in a biosafety level II laboratory is not permitted. Therefore, a pseudovirus-based neutralizing assay is required to evaluate the neutralizing potency of vaccinated mouse sera. Viral entry is dependent on membrane fusion mediated by the spike protein on the envelope of SARS-CoV-2 after its recognition and binding to hACE2 on host cells [45]. Replacing the VSV glycoprotein with the spike protein can imitate the viral entry process while ensuring safety [46]. Different spike expression vectors with mutations in the receptor binding domain (RBD) were constructed and their expression was verified by WB (Figure S1b). The expressed spikes with RBD mutations induced syncytia formation in hACE2 overexpressed 293T monoclonal cells, indicating that the constructed spikes functioned well in mediating membrane fusion (Figure S1c).

**Figure 3:**
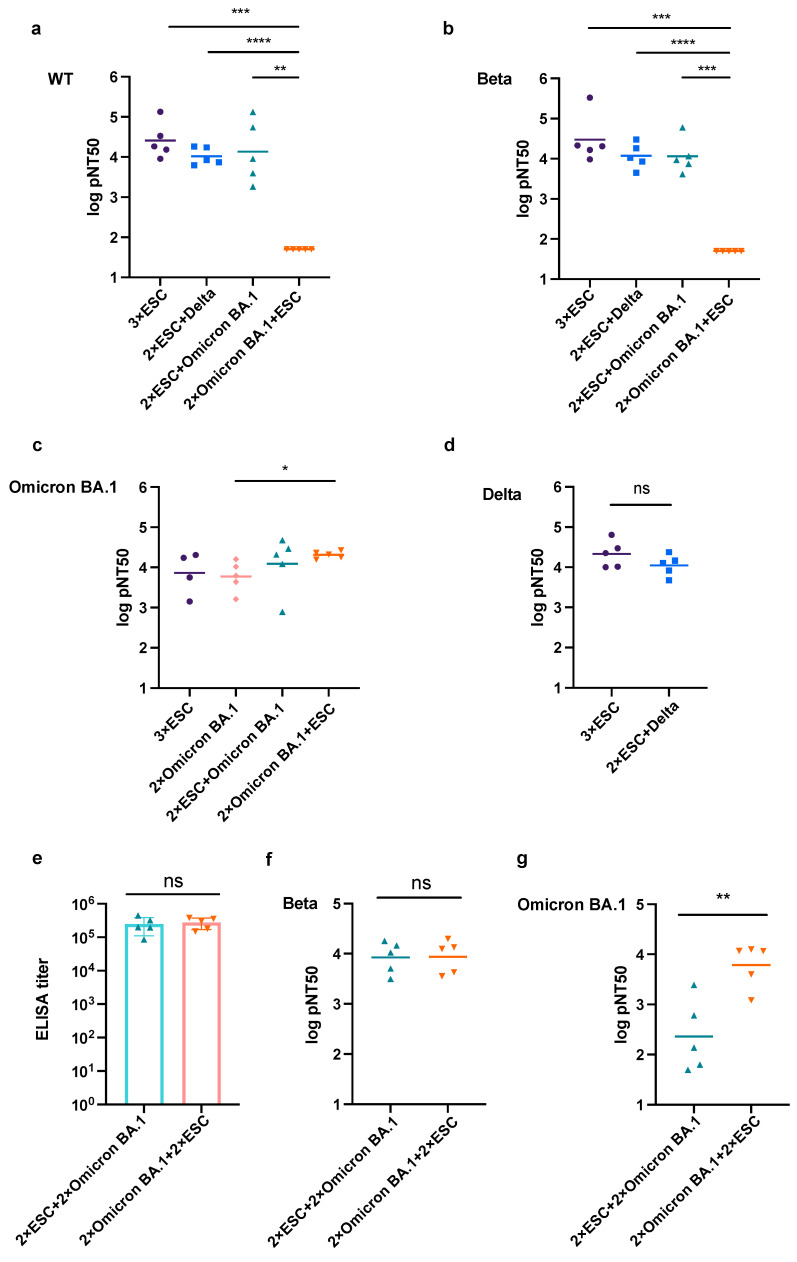
Neutralizing assay of mice sera from different groups against variant pseudoviruses. (**a**,**b**) Common logarithm of 50% pseudovirus neutralization (log pNT50) of triple vaccinated sera of groups A, B, C, and D against wild-type (**a**) and Beta (**b**) pseudoviruses. (**c**) Log pNT50 of triple vaccinated sera of groups A, C, D, and double-vaccinated sera of group D (2×Omicron BA.1) against Omicron BA.1 pseudovirus. (**d**) Log pNT50 of triple vaccinated sera from groups A and B against Delta pseudovirus. (**e**) ELISA titers of the anti-spike antibody in quadruple-vaccinated sera from groups C and D. The endpoint cutoff was determined by Mean_NC_ + 3SD, which was 0.241. (**f**,**g**) Log pNT50 of quadruple-vaccinated sera from groups C and D against Beta (**f**) and Omicron BA.1 (**g**) pseudoviruses. All data are presented as mean ± SD. The difference among different experimental groups were determined by Welch’s test. * *p* < 0.05, ** *p* < 0.01, *** *p* < 0.001, and **** *p* < 0.0001. *n* = 5.

According to the result of pseudovirus-based neutralizing assay, triple-vaccinated sera collected from groups A, B, and C showed a strong neutralizing capacity against wild-type and Beta pseudoviruses, while that from group D in which mice initially received two doses of Omicron BA.1 antigen followed by one dose of ESC showed little neutralizing potency against them (Figure 3a,b). Robust neutralizing potency against Omicron BA.1 pseudovirus was induced by any combination of ESC and Omicron BA.1, and an ESC boost after two doses of Omicron BA.1 significantly increased the neutralizing titer against the Omicron BA.1 pseudovirus (Figure 3c). Mice in group A, which received three doses of the ESC vaccine, and group B, which received two doses of ESC as the prime vaccination and were boosted with Delta, produced equivalent nAbs against the Delta pseudovirus (Figure 3d).

In the neutralization assay of triple-vaccinated sera, groups C and D showed very different neutralization potencies. To rule out the influence of the dosage of different vaccines, we boosted the mice in group C with another dose of Omicron BA.1 and the mice in group D with another dose of ESC to ensure the same dosage and vaccination time interval in both groups. As expected, the same ELISA titer of the two groups was achieved (Figure 3e). There was no significant difference in the nAb titers against the Beta pseudovirus (Figure 3f). However, the mouse sera of group C showed a much lower neutralizing titer against Omicron BA.1 than that of group D (Figure 3g). Therefore, we concluded that the initial vaccination has a greater influence on the neutralizing tendency than booster vaccination, especially when the antigen load was low.

### 3.4. Monoclonal bnAb Can Be Acquired by the Hybridoma Technique from mRNA Immunized Mice

**Figure 4:**
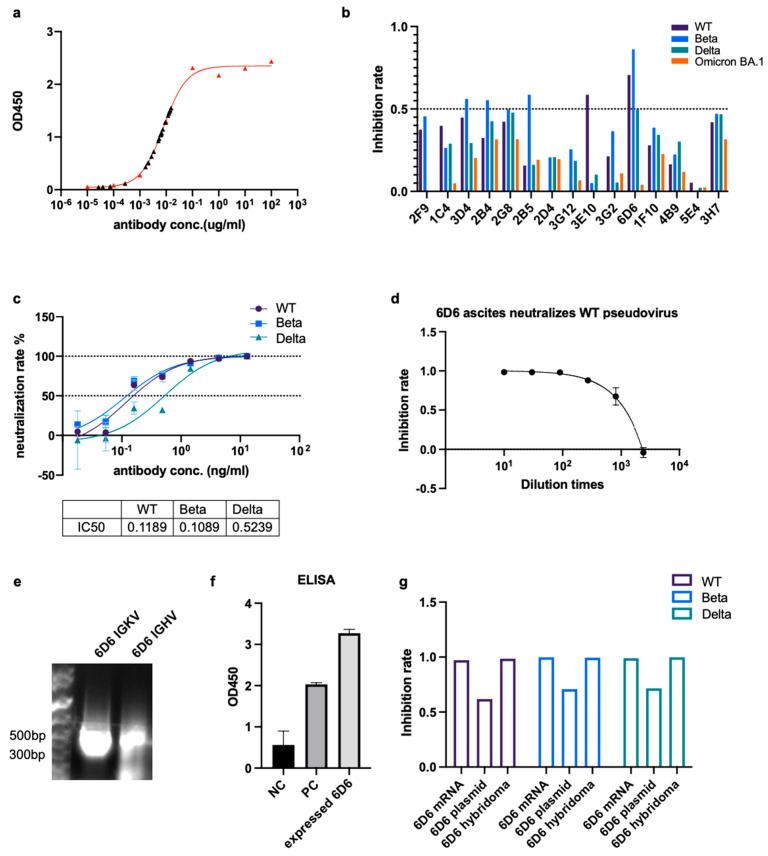
Acquisition and verification of monoclonal broadly neutralizing antibody (nAb). (**a**) OD450 values of ELISA with a standard sample at different concentrations (red triangles) and positive hybridoma media (black triangles). (**b**) Inhibition rate of hybridoma supernatants against wild-type, Beta, Delta, and Omicron BA.1 pseudoviruses. (**c**) Neutralizing rate (%) of the 6D6 monoclonal nAb to wild-type, Beta, and Delta pseudoviruses. The IC50s of 6D6 nAb against wild-type, Beta, and Delta pseudoviruses are 0.1189, 0.1089, and 0.5239 ng/μL, respectively. (**d**) Inhibition rate of ascites culturing the 6D6 hybridoma against wild-type pseudovirus. (**e**) The bands of amplified variable region of kappa chain (6D6 IGKV) and heavy chain (6D6 IGHV) on the agarose gel. (**f**) OD450 values of the ELISA with the medium of 293T cells transfected with expressing vectors of the 6D6 antibody. NC, the medium of untreated 293T cells, and PC, a clinically used SARS-CoV-2 nAb. (**g**) Inhibition rate of the media of 293T cells transfected with 6D6 plasmid, mRNA and 6D6 hybridoma medium to wild-type, Beta and Delta pseudoviruses. The inhibition rate and neutralizing rate are determined by the relative reduction of RLU in neutralizing assay.

To obtain monoclonal antibodies (mAbs) with a broad neutralizing potency, we selected two mice with high titers of nAbs from group C and boosted them with a final dose of ESC through the tail vein. We used the hybridoma technique to obtain mAbs, and we produced dozens of hybridoma cell lines that secrete anti-spike antibodies (Figure S3). The antibody concentrations in the most positive supernatants were 0.001–0.1 μg/mL (Figure 4a). We tested the neutralizing potency of the hybridoma supernatants against wild-type, Beta, Delta, and Omicron BA.1 pseudoviruses. Among all the mAbs, the 6D6 antibody showed a broad neutralizing potency against wild-type, Beta, and Delta pseudovirses (Figure 4b). The IC50s of the 6D6 antibody against the wild-type, Beta, and Delta pseudoviruses were 0.1189, 0.1089, and 0.5239 ng/mL, respectively (Figure 4c). We cultured the 6D6 hybridoma cells in mouse ascites and found that the ascites had potent neutralizing potencies against the wild-type pseudovirus (Figure 4d). We conducted 5′ RACE PCR to amplify the variable region of the heavy and kappa chains, cloned the variable regions into circular vectors with human IgG or IgK constant regions, and sequenced them (Figure 4e). We aligned the sequence of variable regions on the heavy and kappa chains of the 6D6 antibody with other antibody sequences in IMGT/V-QUEST and found that the complementary determining region 3 (CDR3) of the 6D6 antibody was longer than that of other antibodies, indicating its neutralizing potency (Figures S4 and S5) [47]. The anti-spike antibody was detected in 293T cells transfected with vectors expressing the 6D6 antibody (Figure 4f). Finally, we synthesized the mRNA of the heavy chain and the light chain of the 6D6 antibody and verified its neutralizing potency against wild-type, Beta, and Delta pseudovirus of the expressed antibody (Figure 4g).

## 4. Discussion

The COVID-19 pandemic remains a serious issue, and new mutant strains made it more challenging to develop vaccines and effective treatments. However, IVT mRNA technology has advantages in coping with the high mutation rate of the virus, allowing for the rapid development of nAbs against mutant strains. This approach has the potential to produce broad-spectrum neutralizing antibodies targeting various coronaviruses. In this study, we screened and isolated a highly effective nAb that targets multiple SARS-CoV-2 variants using an IVT-mRNA immune strategy.

The protection provided by mAbs primarily targets the NTD and RBD epitopes in the S1 subunit, but this could lead to immune escape by virus mutants [48,49,50]. Antibodies targeting the S2 subunit have a greater probability of being broad-spectrum antibodies to SARS-CoV-2 and other human coronaviruses (HCoVs) [51]. The conserved fusion peptide region adjacent to the S2′ cleavage site is worth exploring as a target for novel vaccines and therapeutics [52,53]. Recent studies by Low et al. and Dacon et al. found that antibodies isolated from convalescent individuals target a conserved fusion peptide region of the viral spike protein, showing broad neutralizing activity against a range of coronaviruses, including the Omicron BA.1 variant of SARS-CoV-2 [54,55]. However, these antibodies have lower potency than RBD-specific antibodies, and there is a trade-off between potency and breadth [31,56].

Different approaches, such as creating libraries of antibody sequences with different combinations of variable regions or using nanobodies can improve antibody efficacy [57,58]. Bispecific or multispecific antibodies that can bind to multiple epitopes simultaneously and combine different antibodies targeting different epitopes may be a potential strategy against COVID-19 [59,60]. The synergistic enhancement of antibodies can lead to better neutralization and protection. Some antibody cocktails have also been explored [61]. In our study, by immunization with chimeric spike mRNA, we obtained mAb clone 6D6 against wild-type, Beta, and Delta variants. The combination of 6D6 with mAbs against the Omicron BA.1 variant may be more effective for combating COVID-19 and enhancing pandemic preparedness.

Similar to our study, other chimeric mRNA-LNP vaccines have been developed based on chimeras of the viral spike proteins composed of domain modules from various epidemic and pandemic coronaviruses as well as bat coronaviruses [62,63]. The idea of a universal vaccine remains attractive because the virus can continue to mutate and render existing vaccines ineffective, and this concept can be applied to provide immunity against viruses that cause different diseases.

Several findings suggest that using a heterologous (adenovirus-based/mRNA, or inactivated vaccine/mRNA) prime-boost regimen schedule could be highly effective in preventing COVID-19 as it leads to a stronger immune response than using the same vaccine for both doses [64]. A previous study suggested that a heterologous boosting schedule could provide enhanced protection against the virus with some improvement in the durability of the response [65,66]. Different variants have unique sets of mutations in the spike protein, resulting in distinct pools of epitopes that are presented to lymphocytes by antigen-presenting cells. Our study supports that sequential immunization may have advantages in inducing broadly neutralization antibodies; however, further investigation is required. Consistent with other studies, we found that variant-specific mRNA vaccines provided the strongest immunity against individual variants. In addition, prior immunity from vaccination may remain effective after boosters and has more influence on the neutralizing tendencies.

The concept of using mRNA encoding for antibodies instead of mAb proteins was first proposed in 2008 (EP 2101823 B1) and the feasibility of passive vaccination with mRNA encoding antibodies was demonstrated in 2017 [67,68]. The mRNAs encoding bispecific Abs were developed to eliminate the advanced tumors and the local delivery of mRNA antibodies is desirable for respiratory viral infections [69,70]. An mRNA (mRNA-1944) encoding a neutralizing antibody against the chikungunya virus has entered a phase 1 trial [71]. In this study, we verified the neutralizing potency of the IVT mRNA encoding the 6D6 antibody, providing a reference for mRNA antibody development.

The advantages of IVT mRNA are that it can effectively cope with the frequent mutations of the coronavirus in time, making it possible to rapidly develop nAbs against mutant strains or potential outbreaks of infectious diseases rapidly. Our study provides some insights into mRNA-dependent bnAbs development, which will shorten the time required for antibody development.

## 5. Conclusions

In summary, we have successfully developed a broadly neutralizing antibody against the wild-type, Beta, and Delta SARS-CoV-2 pseudoviruses and synthesized the IVT mRNA of this antibody. This allowed us to produce bnAbs through mRNA vaccination and IVT mRNA encoding antibodies in a biosafety level II laboratory, effectively shortening the development time of bnAbs. Additionally, our findings suggest that the use of chimeric antigens and sequential immunization has the potential to induce bnAbs, providing valuable insights for vaccine development and the design of immunization strategies.

## Figures and Tables

**Table 1:** Immunization strategy in different groups.

Days	Group A	Group B	Group C	Group D	NC Group
0	ESC high	ESC high	ESC high	Omicron BA.1	Empty LNPs
14	ESC high	ESC high	ESC high	Omicron BA.1	Empty LNPs
28	ESC high	Delta	Omicron BA.1	ESC high	Empty LNPs
42	None	Delta	Omicron BA.1	ESC high	Empty LNPs

## Data Availability

The data supporting the findings of this study are available from the corresponding author upon reasonable request.

## Supplementary Materials

The following supporting information can be downloaded at: https://www.mdpi.com/article/10.3390/pharmaceutics15051412/s1, Figure S1: Spike-mediated membrane fusion experiment; Figure S2: Pseudoviruses preparation and establishment of VSV-based neutralizing assay; Figure S3: Overview of hybridoma technology workflow; Figure S4: Sequence alignment of variable regions of 6D6 heavy chain; Figure S5: Sequence alignment of variable regions of 6D6 kappa chain. Table S1: Primers for mutation induction on spike protein; Table S2: Primers for antibody variable region sequencing and amplification. The sequences of variable regions of heavy and light chains of 6D6 neutralizing antibody were also shown in the supplementary material.
