# Supplementary material for: The Screening of Broadly Neutralizing Antibodies Targeting the SARS-CoV-2 Spike Protein by mRNA Immunization in Mice
Source: Pharmaceutics. 2023 May 5;15(5):1412. doi: 10.3390/pharmaceutics15051412 (PMC10221228; doi:10.3390/pharmaceutics15051412)
Supplement: Supplementary file 1 [file pharmaceutics-15-01412-s001.zip › pharmaceutics-2340874-supplementary.pdf]

1. Supplementary figures:

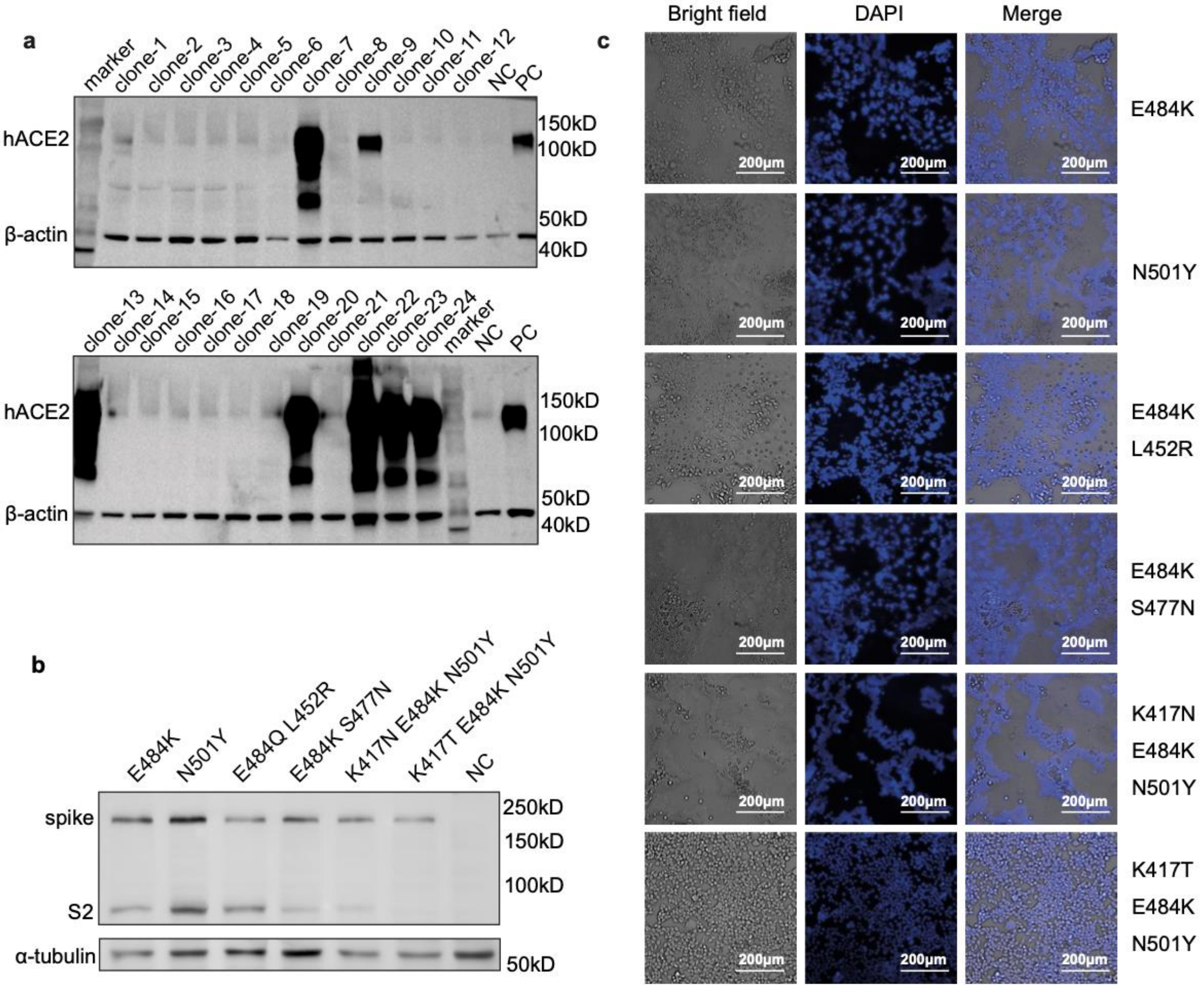

Figure S1. Spike-mediated membrane fusion experiment. (a) Quantification of hACE2 expression in monoclonal 293T cells by WB after stable cell line construction. NC, wild-type HEK293T cells. PC, 293T ACE2 stable cell line constructed by infection of plenti-virus carrying plenti-hACE2-puromycin plasmid and selected by puromycin. (b) Verification of expression of variant spikes introduced specific mutations of in RBD in 293T cells by WB. NC, 293T cells without transfection.

(c) Membrane fusion phenomenon under bright field and fluorescence. Expression vectors of variant spikes were transfected into 293T ACE2 cells. 24 hours later, DAPI was used to stain DNA to observe the syncytia formation. Scale bar, 200 μm.

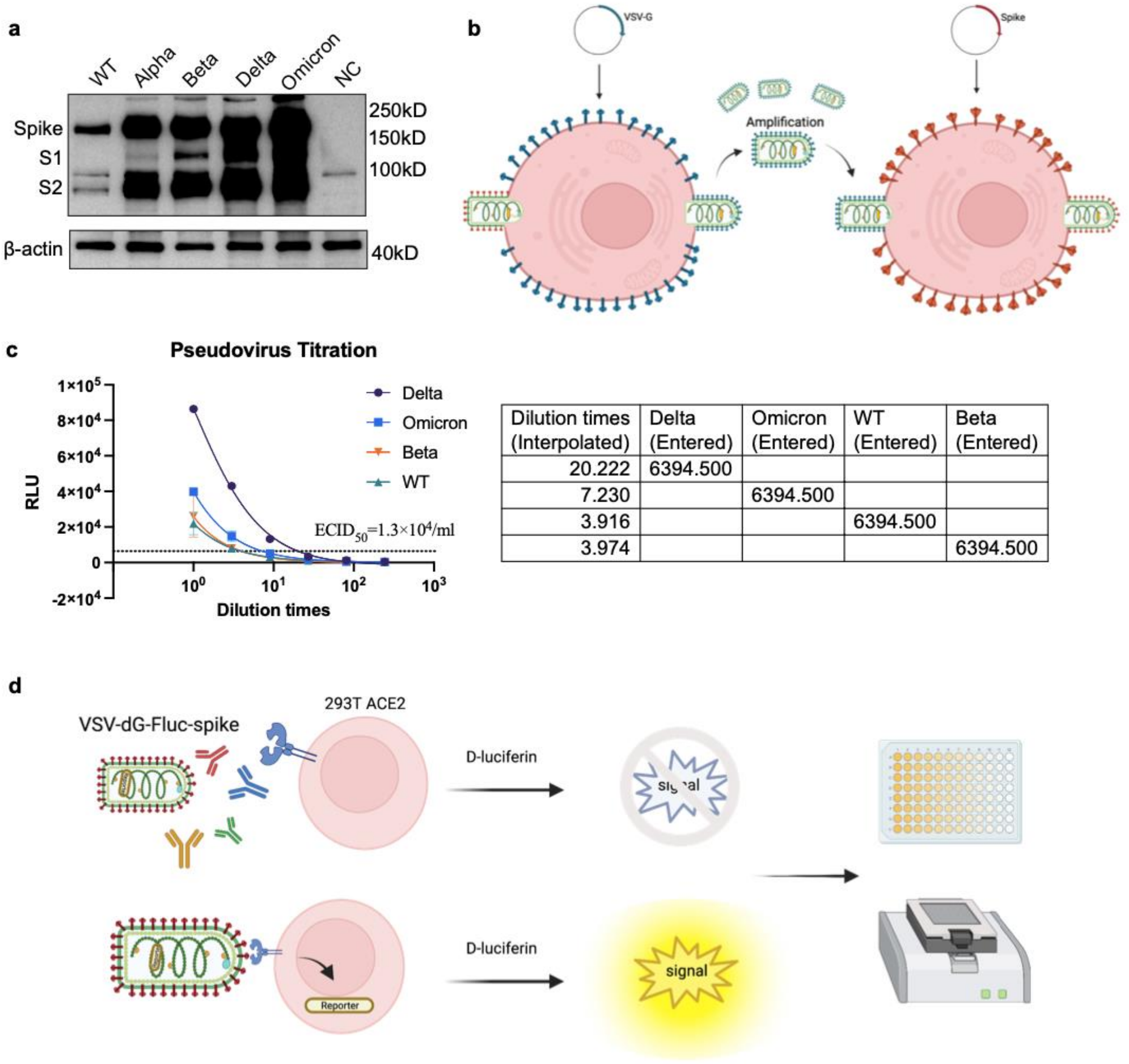

Dilution times (Interpolated)	Delta (Entered)	Omicron (Entered)	WT (Entered)	Beta (Entered)
20.222	6394.500			
7.230		6394.500		
3.916			6394.500	
3.974				6394.500

Figure S2. Pseudoviruses preparation and establishment of VSV-based neutralizing assay. (a) Verification of spike variants expression by WB for pseudoviruses preparation. (b) Diagram of pseudovirus preparation. VSV-dG-Fluc was firstly produced from Omicron BA.1 pseudovirus. The variant pseudoviruses were produced by VSV-dG-Fluc infection following the transfection of expressing vectors of variant spike. The schematic was created on Biorender.com. (c) Pseudovirus titration and dilution times standardized to 1.3×10^4/ml $ECID_{50}$. Titration curves of different variants were on the left while titration times standardized to 1.3×10^4/ml $ECID_{50}$ were shown on the right. (d) Workflow of neutralizing assay. The diagram was created on Biorender.com.

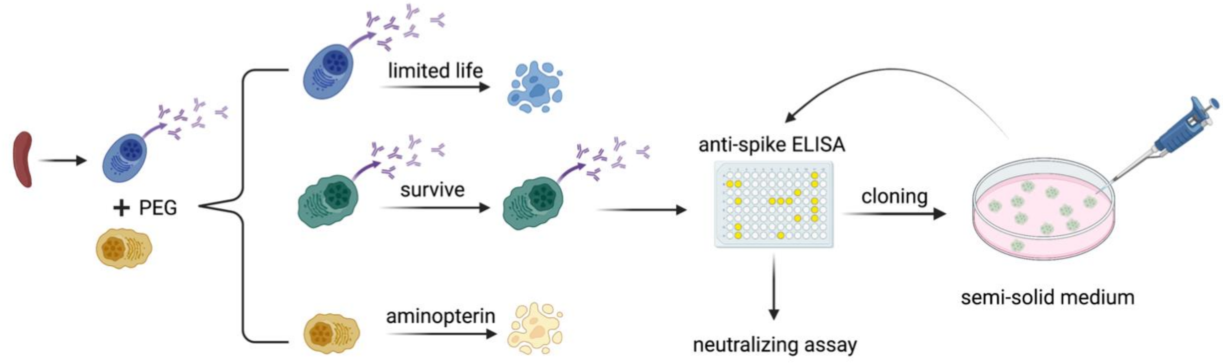

Figure S3. Overview of hybridoma technology workflow. The splenocytes were firstly collected and fused with prepared SP2/0 cells mediated by PEG. After 7-10 days of HAT screening, the positive wells were spotted in ELISA. Semi-solid HT medium was used for cloning of polyclonal cells in positive wells. The picture was created on Biorender.com.

```
                                      <---------------------------------- FR1-IMGT ---------------
M29672_IGHV_6D6                       caggtgcaactgcagcagtctgggcct...cagctggttaggcctggggcttcagtgaag
AF304545 Musmus IGHV1S126*01 [F]      ------------------c-----g--...g-------g-a-------------------
AF304546 Musmus IGHV1S127*01 [F]      -----c------------c-----g--...g-------g-a-------------------
AC160473 Musmus IGHV1-74*01 F         -----c------------c-----g--...g-a-----g-a-------------------
J00537 Musmus IGHV1-74*04 F           --t--c------------c-----g--...g-------g-a-------------------
AC087166 Musmus IGHV1-59*01 F         -----c------------c-----g--...g-------g---------a-----------

                                      ----------------->______________ CDR1-IMGT ___________<-----
M29672_IGHV_6D6                       atatcctgcaaggcttctggttactcattc............accagctactggatgcac
AF304545 Musmus IGHV1S126*01 [F]      --------------------c---a-c---............---------------a--
AF304546 Musmus IGHV1S127*01 [F]      --g-----------------c---a-c---............------------------
AC160473 Musmus IGHV1-74*01 F         g-g-----------------c---a-c---............------------------
J00537 Musmus IGHV1-74*04 F           g-g-----------------c---a-c---............------------------
AC087166 Musmus IGHV1-59*01 F         t-g-----------------c---a-c---............------------------

                                      --------------- FR2-IMGT ------------------->___________ CDR
M29672_IGHV_6D6                       tgggtgaagcagaggcctggacaaggtcttgagtggattggcatgattgatccttcc...
AF304545 Musmus IGHV1S126*01 [F]      --------------------------c-----------c--aga---c--------t...
AF304546 Musmus IGHV1S127*01 [F]      --------------------------c-----------c--a-c------------a...
AC160473 Musmus IGHV1-74*01 F         --------------------c-----c--------------a-g----c-------t...
J00537 Musmus IGHV1-74*04 F           --------------------c-----c--------------a-g----c-------t...
AC087166 Musmus IGHV1-59*01 F         -----a--------------------c-----------c--ag-------------t...

                                      2-IMGT ________<--------------------------------------------
M29672_IGHV_6D6                       ...gatagtgaaactaggttaaatcagaagttcaag...gacaaggccacattgactgta
AF304545 Musmus IGHV1S126*01 [F]      ...------t-t----acaac-----a---------...---------------------
AF304546 Musmus IGHV1S127*01 [F]      ...------t-t-----c-ac-----a---------...-g-------------------
AC160473 Musmus IGHV1-74*01 F         ...--------t----ac-ac-----a---------...-g-------------------
J00537 Musmus IGHV1-74*04 F           ...--------t----ac-ac-----a---------...-g-------------------
AC087166 Musmus IGHV1-59*01 F         ...------t-t----ac-ac-----a---------...-g-------------------

                                      --------- FR3-IMGT -----------------------------------------
M29672_IGHV_6D6                       gacaaatcctccagcacagcctacatgcaactcagcagcccgacatctgaggactctgcg
AF304545 Musmus IGHV1S126*01 [F]      -----------------------------g----------t-------------------
AF304546 Musmus IGHV1S127*01 [F]      ----c------------------------g----------t---------a---------
AC160473 Musmus IGHV1-74*01 F         -----------------------------g----------t-------------------
J00537 Musmus IGHV1-74*04 F           -----------------------------g----------t-------------------
AC087166 Musmus IGHV1-59*01 F         ----c------------------------g----------t-------------------

                                      ----------->___________ CDR3-IMGT ________
M29672_IGHV_6D6                       gtctattactgtgcaagagggggggaccctaactttgactactggggccaaggcaccacg
AF304545 Musmus IGHV1S126*01 [F]      ------------------
AF304546 Musmus IGHV1S127*01 [F]      ------------a-----
AC160473 Musmus IGHV1-74*01 F         ----------------t-
J00537 Musmus IGHV1-74*04 F           ----------------t-
AC087166 Musmus IGHV1-59*01 F         ------------------
```

Figure S4. Sequence alignment of variable regions of 6D6 heavy chain. The antibody alignment was completed on IMGT/V-QUEST. The variable region of 6D6 heavy chain was named as M29672_IGHV_6D6.

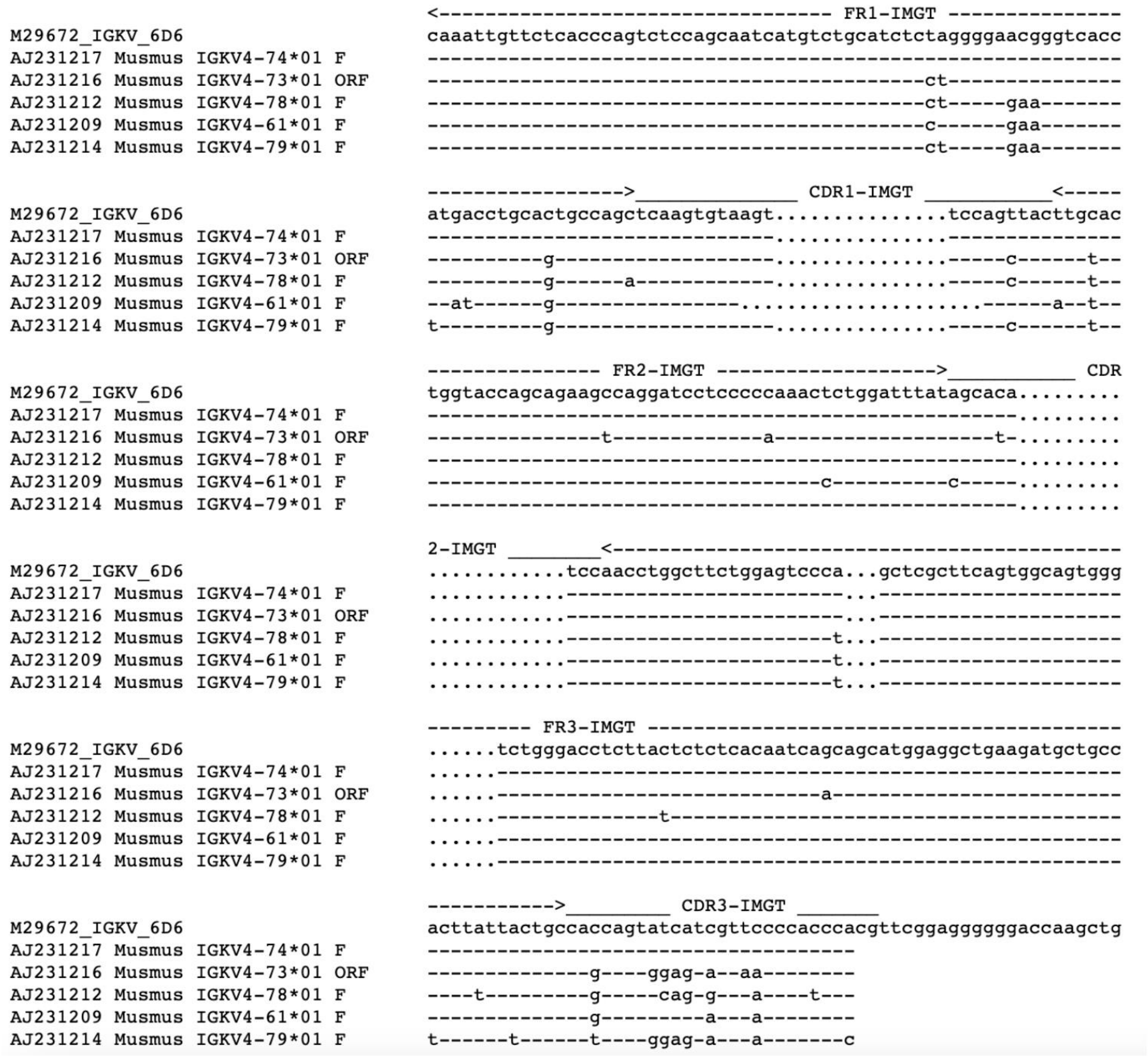

Figure S5. Sequence alignment of variable regions of 6D6 kappa chain. The antibody alignment was completed on IMGT/V-QUEST. The variable region of 6D6 kappa chain was named as M29672_IGKV_6D6.

2. Supplementary tables:

Table S1. Primers for mutation induction on spike protein.

Primers	Sequence
T478K-F	CCGGCAGCAAACCTTGCAAT
T478K-R	ATTGCAAGGTTTGCTGCCGG

P681R-F	GCAGAAGAGCCCGGAGC
P681R-R	CGAGAGTTTGTCTGGGTCTGG
D950N-F	CAAGCTCCAGAATGTGGTGAATC
D950N-R	GATTCACCACATTCTGGAGCTTG
E156G Del157/158-F	GGAGTCCGGGGTGTATTCTAGCGCCAACAACTGC
E156G Del157/158-R	TAGAATACACCCCGGACTCCATCCAGCTCTTATTG
T19R-F	CGTCAATCTGAGAACTCGGACTC
T19R-R	GAGTCCGAGTTCTCAGATTGACG
A701V-F	CTGGGCGTCGAGAACAG
A701V-R	CTGTTCTCGACGCCCAG
D80A-F	AGCGGTTCGCCAATCCCG
D80A-R	CGGGATTGGCGAACCGCT
D215G-F	GTGCGCGGCCTGCCT
D215G-R	AGGCAGGCCGCGCAC

Table S2. Primers for antibody variable region sequencing and amplification.

Primers	Sequence
Template-switch oligo	AAGCAGTGGTATCAACGCAGAGTACATGrGrGr
mIGK RT	TTGTCGTTCACTGCCATCAATC
mIGL RT	GGGGTACCATCTACCTTCCAG
mIGHG RT	AGCTGGGAAGGTGTGCACAC

ISPCR	AAGCAGTGGTATCAACGCAGAG
mIGK PCR	ACATTGATGTCTTTGGGGTAGAAG
mIGL PCR	ATCGTACACACCAGTGTGGC
mIGHG PCR	GGGATCCAGAGTTCCAGGTC
mouse IgG nest-2 R	CCTTGGTGGAGGCGTCGAACGGTGACCGTGG
	CCTTGGTGGAGGCGTCGAACTGTGAGAGTGG
	CCTTGGTGGAGGCGTCGAACAGTGACCAGAG
	CCTTGGTGGAGGCGTCGAACGGTGACTGAGG
mouse IgK nest-2 R	GCCGCCACAGTTCGTCGATTTCAGCTCCAGCTTGGTCCC
	GCCGCCACAGTTCGTCGATTTTATTTCCAGTCTGGTCCC
	GCCGCCACAGTTCGTCGATTTKATTTCCARCTTKGTSCC
cloning-ISPCR-F	ACAGGAGCCCACTCCCGTACAAGCAGTGGTATCAACGCAGAG
K/L-cloning-ISPCR-F	CCAGGTTCCAGATGCACCGGAAGCAGTGGTATCAACGCAGAG

3. Antibody sequence：

Variable region of heavy chain of 6D6 neutralizing antibody:

CAGGTGCAACTGCAGCAGTCTGGGCCTCAGCTGGTTAGGCCTGGGGCTTCAGTGAAGAT
ATCCTGCAAGGCTTCTGGTTACTCATTCACCAGCTACTGGATGCACTGGGTGAAGCAGA
GGCCTGGACAAGGTCTTGAGTGGATTGGCATGATTGATCCTTCCGATAGTGAAACTAGG
TTAAATCAGAAGTTCAAGGACAAGGCCACATTGACTGTAGACAAATCCTCCAGCACAGC
CTACATGCAACTCAGCAGCCCGACATCTGAGGACTCTGCGGTCTATTACTGTGCAAGAG
GGGGGGACCCTAACTTTGACTACTGGGGCCAAGGCACCACGGTCACCGTCTCG

Variable region of light chain of 6D6 neutralizing antibody:

CAAATTGTTCTCACCCAGTCTCCAGCAATCATGTCTGCATCTCTAGGGGAACGGGTCACC
ATGACCTGCACTGCCAGCTCAAGTGTAAGTTCCAGTTACTTGCACTGGTACCAGCAGAA

GCCAGGATCCTCCCCCAAACTCTGGATTTATAGCACATCCAACCTGGCTTCTGGAGTCCC
AGCTCGCTTCAGTGGCAGTGGGTCTGGGACCTCTTACTCTCTCACAATCAGCAGCATGGA
GGCTGAAGATGCTGCCACTTATTACTGCCACCAGTATCAT
